# 5′-UMP inhibited muscle atrophy due to detraining: a randomized, double-blinded, placebo-controlled, parallel-group comparative study

**DOI:** 10.3389/fspor.2024.1403215

**Published:** 2024-07-15

**Authors:** Mika Inoue, Atsushi Kubota, Yuji Takazawa, Kosuke Nakagawara, Kazuya Ishige, Yoshio Suzuki

**Affiliations:** ^1^ Juntendo Administration for Sports, Health and Medical Sciences, Juntendo University, Tokyo, Japan; ^2^ Graduate School of Health and Sports Science, Juntendo University, Chiba, Japan; ^3^ Graduate School of Medicine, Juntendo University, Tokyo, Japan; ^4^ Biochemicals Division, YAMASA Corporation, Chiba, Japan

**Keywords:** muscle thickness, resistance training, sarcopenia, UMP, uridine 5′-monophosphate

## Abstract

**Purpose:**

A short period of disuse of 1–2 weeks due to factors such as illness or injury can lead to muscle atrophy, affecting both athletic performance and health. Recent research has shown that uridine 5′-monophosphate (5′-UMP) can counteract disuse-induced muscle atrophy by increasing PGC-1α expression and inhibiting atrogin-1 expression. However, the effect of 5′-UMP on disuse muscle atrophy in humans remains unknown. Therefore, the aimed of this study was to explore the effects of 5′-UMP supplementation during detraining on short-term disuse muscle atrophy in healthy men.

**Methods:**

Following a 6-week resistance training program on upper arm, healthy men were randomized to either a UMP group (*n* = 11) or a placebo group (*n* = 10), taking their respective supplements during the 2-week detraining period. Muscle thickness, an indicator of muscle hypertrophy and atrophy, was measured at 3 positions (MT50, MT60, and MT70) at baseline, 1 week, and 2 weeks after detraining.

**Results:**

Both groups showed a significant decrease in muscle thickness at MT70. The relative decrease was greater in the placebo group (2.4 ± 2.8%) than in the UMP group (0.0 ± 2.0%), significantly (*p* = 0.034) at 1 week. However, no significant difference was observed at MT50 and MT60.

**Conclusion:**

After the hypertrophy, 5′-UMP may prevent muscle atrophy due to the detraining within the first week.

## 1 Introduction

Skeletal muscle mass reduction occurs in short- and long-term situations of muscle disuse, such as during illness or injury, a condition referred to as “disuse muscle atrophy.” This phenomenon adversely affects health and results in a loss of muscle function (1). The onset of disuse muscle atrophy can be triggered by as little as 2 days of muscle inactivity (2, 3), progressing rapidly within a 2-week timeframe (4, 5). In older individuals, the average hospitalization duration for acute illness is 5–6 days (6). The reduced physical activity and muscle function during this brief hospital stay are associated with an increased risk of subsequent muscle function decline and mortality (7, 8). Athletes, too, face periods of reduced physical activity, such as 1–2 weeks of joint immobilization after common sports injuries like anterior cruciate ligament surgery (9, 10), or a week of decreased activity following an ankle sprain (11). Studies have reported that athletes experience muscle atrophy and diminished muscle function after just 2 weeks of training cessation (detraining) (12–15). To expedite their return to play, athletes must minimize muscle atrophy and loss of muscle function during short-term detraining. Although resistance training is recognized as effective in preventing disuse muscle atrophy (16), its application during periods of illness or injury can be challenging. Therefore, a nutritional strategy, without exercise intervention, becomes essential for preventing disuse muscle atrophy induced during short-term illness or injury (1–2 weeks) (1).

The degradation of muscle proteins through the ubiquitin–proteasome system plays a role in the early phase of disuse muscle atrophy progression (17, 18). Specifically, the muscle tissue-specific ubiquitin ligase, atrogin-1, is strongly induced during the onset of muscle atrophy (19), leading to its occurrence (19, 20). Early-phase disuse muscle atrophy is more pronounced in slow-twitch muscle fibers than in fast-twitch ones (21). In this context, the transcriptional cofactor PGC-1α inhibits FoxO3 activity and ubiquitin ligase expression, serving to prevent disuse muscle atrophy (22). Exercise increases PGC-1α, promoting the transformation of fast-twitch muscle fibers into slow-twitch ones (23, 24). Conversely, the expression of PGC-1α decreases with restricted physical activity, such as joint immobilization (25). Thus, preventing the decline in PGC-1α expression and the rise in ubiquitin ligase is crucial for averting early-phase disuse muscle atrophy. Recent findings demonstrate that PGC-1α expression was enhanced (26), and the increased expression of atrogin-1 was inhibited (27) in mouse myoblasts C2C12 after 6 days of differentiation in culture with uridine 5′-monophosphate (5′-UMP), a component of ribonucleic acid. Moreover, 5′-UMP induces an increase in slow-twitch muscle fibers (26) and inhibits the reduction in myotube cell diameter (27). Therefore, 5′-UMP may prevent disuse muscle atrophy by increasing PGC-1α expression and inhibiting atrogin-1 expression. UMP is a type of nucleotide that makes up RNA. The total amount metabolized in an adult exceeds to approximately 750 mg per day. UMP disodium salt (UMP, 2Na) is used as a food additive primarily for seasoning. Previous studies have shown that no adverse effects were not observed in humans taking 1,000 mg/day of 5′-UMP, 2Na for 8 days. However, the preventive effect of 5′-UMP on disuse muscle atrophy in human subjects remains unknown.

A previous study demonstrated muscle atrophy and strength loss, resulting from a 2-week detraining period after muscle hypertrophy via upper extremity resistance training (28). Muscle hypertrophy caused by high-intensity training occurs in both fast-twitch and slow-twitch fibers (29). This study aims to assess the preventive impact of 5′-UMP intake during detraining on short-term disuse muscle atrophy in healthy men. We hypothesized that 5′-UMP intake during detraining will mitigate muscle atrophy.

## 2 Method

### 2.1 Participants

A total of 22 healthy men were enrolled in this study. The inclusion criteria were defined as follows: (1) age exceeding 20 and below 25 years, (2) BMI ≤ 30 kg/m^2^, (3) satisfactory physical condition with the ability to safely perform the experiment, and (4) provision of written consent and voluntary participation in the experiment. Conversely, exclusion criteria comprised (1) regular upper extremity training; (2) presence of glucose metabolism disorders, endocrine disorders, kidney/liver disease, and digestive disease; (3) current medical medication prescribed by a doctor; (4) diagnosed heart disease; (5) type I or type II diabetes; (6) a physical examination within the past year requiring treatment or reexamination; (7) participation in another clinical experiment within the past 3 months; (8) use of illicit drugs within the past 6 months; or (9) a history of alcoholism or drug addiction within the past 6 months. The study's purpose and methods were communicated verbally and in writing to the participants, who then provided written consent.

**Figure 1:**
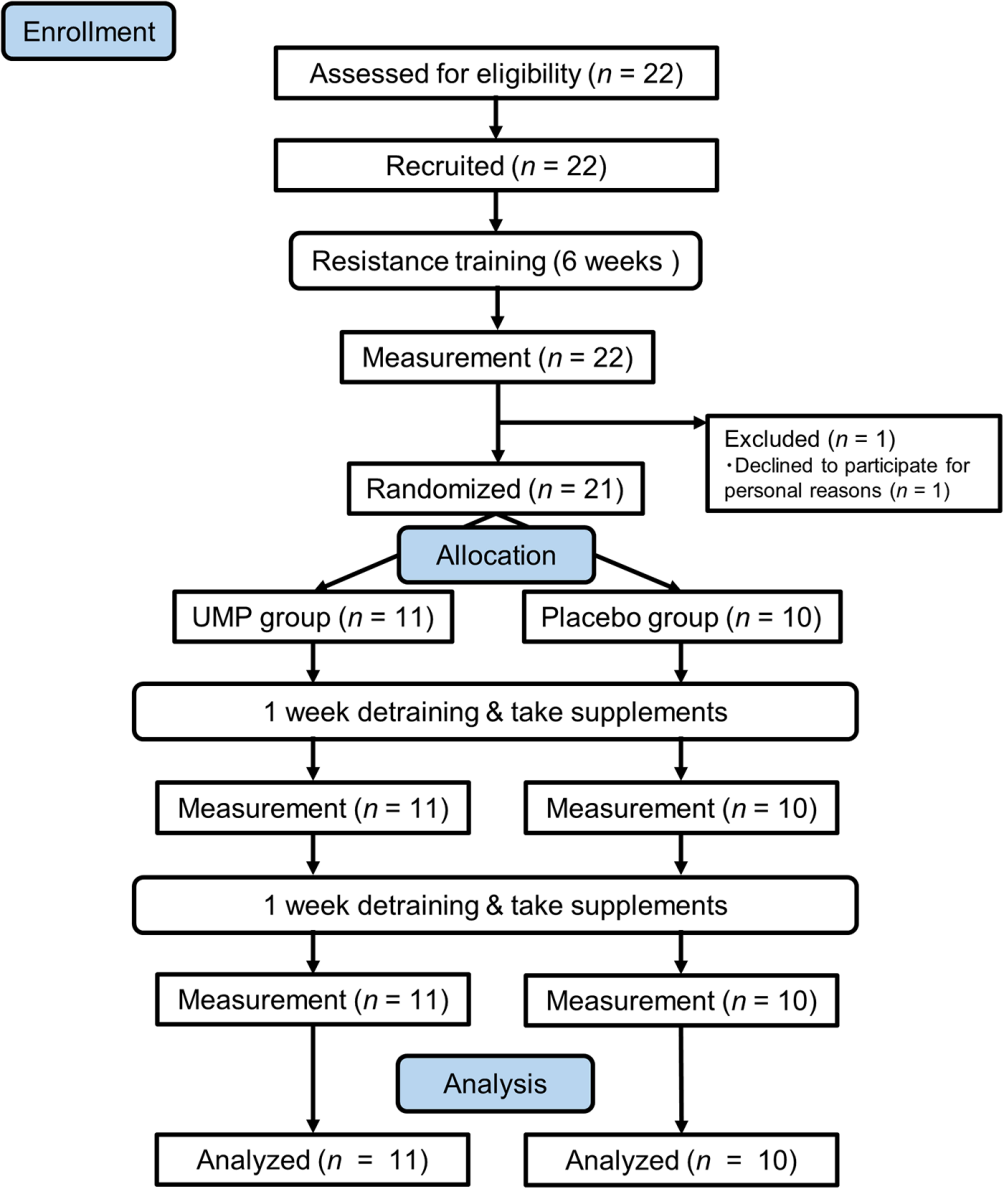
Flow diagram of participants.

One participant opted not to continue for personal reasons before controlled trial. The remaining 21 participants were randomly assigned to two groups: (1) receiving 5′-UMP, 2Na (UMP group, *n* = 11) and (2) a placebo group (*n* = 10), and they were allowed to consume the assigned test supplement during the 2-week detraining phase. Measurements were taken weekly during the detraining period, and data from the 21 participants who completed the study were used for analysis (Figure 1).

The study adhered to the principles of the Declaration of Helsinki and received approval from the Ethics Committee of Juntendo University Graduate School of Health and Sports Science (Approved: #2021-74). Prior to participant recruitment, the study was registered with the UMIN Clinical Trials Registry (UMIN000045069).

### 2.2 Experimental design

This experiment was comprised of 6 weeks of training period and following 2 weeks of detraining period. During the training period, the participants underwent upper extremity resistance training to hypertrophy their upper arms (details described in Resistance training protocol). A randomized, double-blinded, placebo-controlled, parallel-group trial was then conducted during the detraining period to assess the effect of UMP.

Throughout the 2-week detraining period, each participant ingested either 5′-UMP, 2Na or placebo supplements (refer to Test Supplement) once daily before bedtime. Participants were directed to promptly notify a liaison researcher after taking the test supplement for confirmation. In cases where no contact was received, reminders were provided via phone, so the supplement intake rate during detraining was 100% for both the UMP and placebo groups. Participants were instructed to maintain their eating habits and not to engage in upper extremity resistance training during detraining period. Muscle thickness, serving as an indicator of muscle hypertrophy and atrophy, and maximal muscle strength were measured at the beginning (Baseline), 1 week (1 week), and 2 weeks (2 week) of the detraining period (refer to Measurements).

### 2.3 Resistance training protocol

The athletes after injury or surgery as well as elderly population often train under isometric contractions because it is difficult to move the affected part. Thereby, resistance training was conducted following the modified approach outlined by Endo et al. (28), published in Juntendo Medical Journal, the official journal of the Juntendo Medical Society. Each participant engaged in isometric elbow flexion with their non-dominant arm the elbow joint angle fixed at 90° (Supplementary Figure S1) using the Biodex System 4 (Biodex Medical Systems, Inc., Shirley, NY, USA). Throughout the training, participants were seated, and the upper body was secured with a non-stretchable belt, while the shoulder joint was secured with an attached pad in a 45° flexion position and a 30°–40° abduction position. The training regimen consisted of 10 sets of 5-s maximal contractions followed by 5-s rests (1-min rest between sets). Prior to the training, participants were allowed a sufficient warm-up using the Biodex System 4 by performing several submaximal contractions in a similar position to training. Nineteen participants completed 3 sessions per week with at least 48 h of rest between each session for 6 weeks (a total of 18 sessions). Three participants completed 2–4 sessions per week with at least 24 h of rest between each session for 6 weeks (a total of 18 sessions) since the schedule was not met.

### 2.4 Test supplement

A capsule assigned to the UMP group contained 150 mg of 5′-UMP, 2Na and crystalline cellulose as an excipient. In contrast, a capsule given to the placebo group contained only crystalline cellulose. Participants consumed 2 capsules once daily during the detraining period. The capsules were identical in appearance, shape, color, odor, or taste, and each was individually packaged in aluminium bags featuring only an identification (ID) printed on them. Randomization was performed by letting each participant choose an aluminium bag without seeing its ID. The contents of the capsules remained unknown to the researchers and participants until the study completion and data analysis. The supplements were supplied by YAMASA Corporation (Chiba, Japan).

The daily dose of UMP was determined as follows. The continuous administration of two nucleotides, cytidine 5′-monophosphate (3.0 mg/kg) and 5′-UMP (2.5 mg/kg), to rats for 10 days improved endurance exercise performance without any adverse symptoms by administration (30). The total intake of the two nucleotides, 5′-monophosphate (3.0 mg/kg) and 5′-UMP (2.5 mg/kg), was calculated to be 5.5 mg/kg. Based on this result, dosage of the supplement was calculated using the following formula: 5.5 mg/kg × 57 kg = 314 mg, based on the average weight of a 20-year-old Japanese male, which is 57 kg. Consequently, the daily dose to the subjects of this study was determined to be 300 mg.

### 2.5 Measurements

#### 2.5.1 Muscle strength

The maximal voluntary isometric contractions were performed twice for 5 s, replicating the position from the resistance training (refer to Resistance training protocol) with the Biodex System 4. The higher maximum torque, measured in N·m, was used for analysis. Before the actual measurement, participants were given the opportunity to practice the procedure multiple times to become acquainted with it.

#### 2.5.2 Muscle thickness

Muscle thickness was assessed using B-mode elastography (Noblus, Hitachi Aloka Medical, Ltd., Tokyo, Japan) in the biceps and brachialis muscles. The linear probe (EUP-L65; Hitachi Aloka Medical, Ltd.) was gently applied to the muscle without exerting pressure, and muscle thickness was measured in increments of 0.1 mm based on the clear image obtained during the measurement. Measurements were taken at three specific sites: 50%, 60%, and 70% of the distal part of the brachial length (from the acromion to the lateral epicondyle) and at the point of intersection when a perpendicular line was drawn between the acromion and biceps tendon (MT50, MT60, and MT70 as shown in Supplementary Figure S2). Participants were positioned in a supine position on the treatment bed, with the shoulder joint at 20° abduction and 30° flexion, and the forearm in external rotation, using a pad placed under the forearm. During the measurement, participants were instructed to remain relaxed.

The measurements were conducted three times, and the average value was used for subsequent analysis. Ultrasound images obtained at the first measurement served as a reference to ensure consistent measurement positioning across all measurements. Intraclass correlation coefficients [ICCs (1, 3)] and coefficients of variation (CVs) were computed based on the data before the training. The resulting ICCs (95% CI) were as follows: MT50, 1.000 (0.999–1.000); MT60, 0.999 (0.999–1.000); and MT70, 0.999 (0.999–1.000), indicating a high level of reliability, similar to the previous study (31). Furthermore, the CV was 0.3% for MT50, 0.3% for MT60, and 0.3% for MT70.

### 2.6 Statistical analysis

Results are presented as mean ± SD. The normality of the data was assessed using the Shapiro–Wilk test. As normal distribution was not assumed in some cases, the Wilcoxon signed-rank test was used to assess the impact of resistance training. Changes in muscle strength and thickness resulting from supplementation were evaluated using Friedman's test in each group. If a significant difference was observed, the Bonferroni method was used as a *post hoc* test to compare results between each time point. Mann–Whitney *U*-test was employed to compare the percentage changes between groups. The between-group effect size (*r*) was calculated using the following formula: *r* = Z/√n. Effect sizes were classified as small (0.1 ≤ *r* < 0.3), medium (0.3 ≤ *r* < 0.5), or large (0.5 ≤ *r*). All statistical analyses were conducted using SPSS statistical software (version 29.0.0.0; IBM SPSS Statistics Japan Inc.). The significance level was set at *p* < 0.05.

## 3 Results

There were no significantly differences in the age, height, weight, and BMI of the analyzed participants at enrollment for the UMP and placebo groups (Table 1).

**Table 1:** Characteristics of participants before resistance training.

	Placebo (*n* = 10)	UMP (*n* = 11)	*p*
Age (years)	21.5 ± 1.6	21.2 ± 1.5	0.557
Height (m)	1.74 ± 0.07	1.72 ± 0.04	0.605
Body weight (kg)	67.8 ± 11.5	64.5 ± 6.0	0.512
BMI (kg/m^2^)	22.4 ± 3.2	21.7 ± 1.5	0.809

Data are expressed as mean ± SD.

The 6 weeks of resistance training gave a significant increase in muscle strength (*p* = 0.002) and thickness (*p* < 0.001, Supplementary Table S1 in supplementary material).

No differences in muscle strength and thickness were observed between the groups at Baseline (Table 2). During the detraining period, a significant decrease was observed in the muscle thickness at MT70 in UMP and placebo groups, respectively. However, the post-hoc test showed no significant difference between time points. There were no significant changes in muscle strength, and muscle thickness at MT50 and MT60 (Table 2).

**Table 2:** Changes in muscle strength and thickness during the detraining period.

	Before-training	group	Baseline	1 week	2 weeks	*p*
Muscle strength (N·m)	55.2 ± 11.6	Placebo	66.3 ± 16.2	64.0 ± 15.7	64.5 ± 14.8	0.716
		UMP	61.1 ± 9.3	60.9 ± 10.1	60.3 ± 9.2	0.202
Muscle thickness (mm)						
MT50	27.4 ± 3.0	Placebo	29.6 ± 3.3	29.0 ± 3.0	29.0 ± 3.5	0.500
		UMP	28.6 ± 1.8	28.4 ± 2.4	28.3 ± 2.4	0.061
MT60	31.9 ± 3.1	Placebo	34.4 ± 3.5	34.0 ± 3.4	33.6 ± 3.9	0.169
		UMP	33.2 ± 2.2	33.1 ± 2.3	33.0 ± 2.0	0.191
MT70	33.6 ± 3.1	Placebo	36.4 ± 3.5	35.5 ± 3.4	35.5 ± 3.7	0.019*
		UMP	35.4 ± 2.4	35.4 ± 2.4	34.9 ± 2.2	0.015*

Data are expressed as mean ± SD. *p < 0.05 vs. baseline.

**Figure 2:**
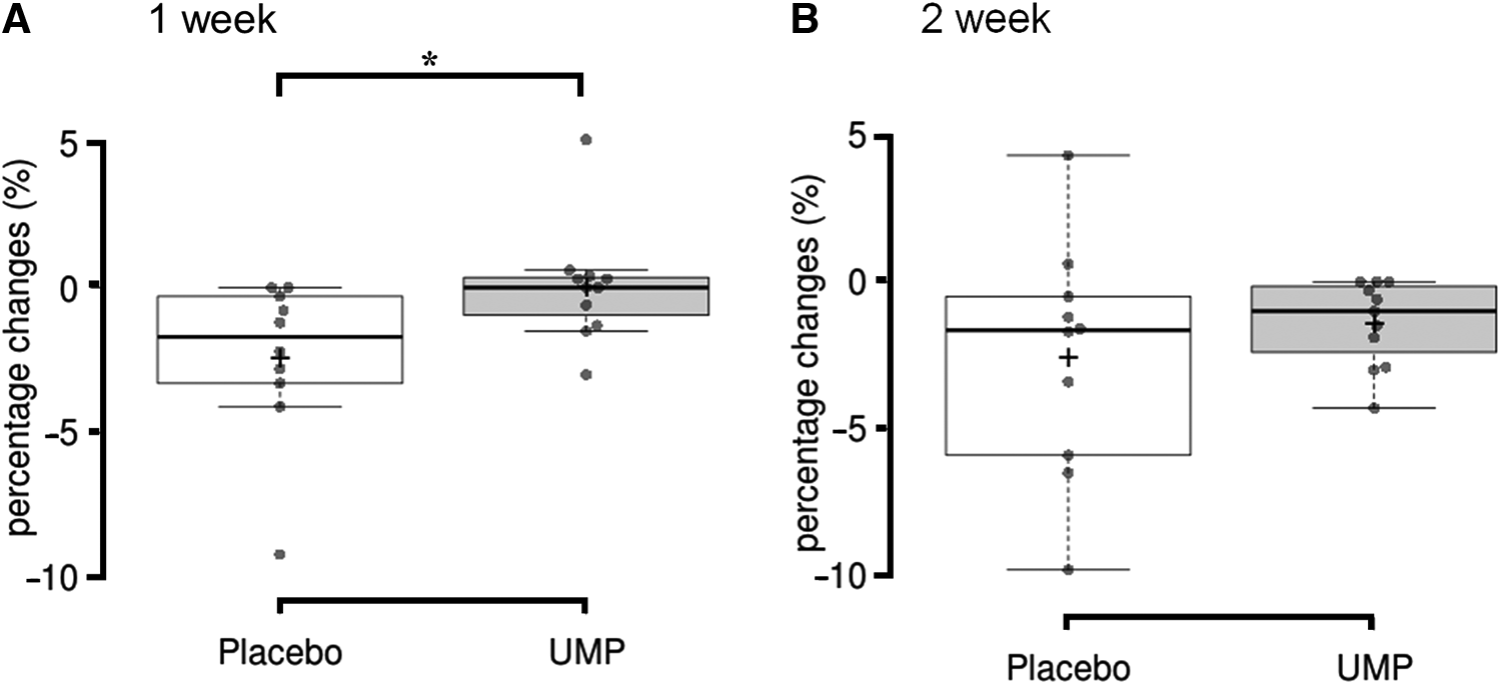
The decrease from baseline to (**A**) 1 week and (**B**) 2 weeks of detraining in muscle thickness. *p < 0.05. ^+^, the group mean value; upper and lower whisker, maximum and minimum values; the line splitting in the box in two, the median value; the upper edge of the box, the upper quartile (i.e., 75% of the data fall below the upper quartile); the lower edge of the box, the lower quartile (i.e., 25% of the data fall below the lower quartile).

The percentage change from baseline in the muscle thickness at MT70 was significantly greater in the placebo group (−2.4 ± 2.8%) compared to the UMP group (0.0 ± 2.0%, *p* = 0.020, *r* = −0.51; Figure 2A) at 1 week. The percentage change in the muscle thickness at MT70 in the UMP group (−1.4 ± 1.5%) was still less than that in the placebo group (−2.6 ± 4.0%) at 2 weeks, although the difference was not significant (*p* = 0.426, *r* = −0.19; Figure 2B). In contrast, there was no significant difference in percentage changes between groups in muscle thickness at MT50 and MT60 at 1 week and 2 weeks.

During the period of this study, no adverse effects caused by the intake of test foods were observed in all participants.

## 4 Discussion

The muscle atrophy rate resulting from 1-week detraining was lower in the group that consumed 5′-UMP, 2Na once a day during detraining (0.0%) compared to the group that did not consume 5′-UMP, 2Na (2.4%). This was observed in only one of three sites in the biceps and brachialis muscles. It is likely that the intake of 5′-UMP during detraining has the potential to prevent muscle atrophy within the first week after detraining. However, there was no observed change in muscle strength during the 2-week detraining period.

A previous study protocol (28) demonstrated that 3 weeks of detraining induced approximately 3.1% muscle atrophy, and this protocol was adopted in the current study. In our investigation, muscle atrophy was induced by approximately 2.6% in the placebo group, a result consistent with the earlier study. Notably, muscle atrophy was specifically observed at MT70. It has been suggested that resistance training at long muscle lengths results in higher tension in the distal region than in other regions (32), leading to greater hypertrophy in the distal region than in other regions (33). During the isometric contraction at a fixed elbow angle of 90°, the elbow flexor muscle shortens in the proximal direction, and a high elongation force is applied to the distal region. This may be the cause that muscle hypertrophy was observed only at the MT70 position. In addition, fast-twitch muscle fibers are recruited by increasing exercise intensity (34). Training-induced adaptations occur in response to the training performed. In this study, participants trained at maximal effort (high intensity). This may have induced more training adaptation in the fast-twitch muscle fibers, and the associated muscle atrophy may have been more obvious in the fast-twitch muscle fibers. MT70 is the most distal measurement site of the biceps brachii. Since there are more fast-twitch muscle fibers near the insertion of the biceps brachii than at the origin (35), it is likely that apparent muscle atrophy was seen only at MT70. However, this is not clear since we have not examined the change in myofiber composition.

Disuse muscle atrophy results from decreased muscle protein synthesis and/or increased protein breakdown (36, 37). Among these factors, there is a rapid decrease in protein synthesis occurs during the first 2 weeks of disuse and a subsequent decline (1, 2, 21, 38). Atrogin-1, a mediator of muscle protein degradation, increases after 2–7 days of disuse (19, 39–41) and returns to baseline after 14 days (40, 42, 43). 5′-UMP has been shown to increase PGC-1α expression (26) and inhibit atrogin-1 expression, thereby preventing the reduction of myotube cell diameter (27). PGC-1α prevents muscle atrophy by inhibiting FoxO3 activity and the expression of atrogin-1 and MuRF1, ubiquitin ligases (22). In light of this evidence, the mechanism underlying these results could be the consumption of 5′-UMP inhibits protein degradation by downregulating atrogin-1 through increased expression of PGC-1α, thereby preventing muscle atrophy within 1 week of detraining in the present study.

Disuse muscle atrophy caused by inactivity occurs in as little as 2 days (2, 3). Athletes may experience muscle atrophy after 2 weeks of training cessation due to injury (12–15). It is important to reduce the muscle atrophy that occurs within a short period following such a training cessation to hasten the return to daily life and competition. The present study showed that in a young sample, UMP intake reduced muscle atrophy in a period as short as 1 week after detraining. Further studies with biomolecular methods on biopsy samples are still needed to verify these results and mechanistic hypotheses. However, 5′-UMP may be beneficial as a strategy to prevent disuse muscle atrophy induced during short-term illness or injury in young men.

On the other hand, it is not clear whether the UMP can be adapted to the elderly population, since we examined the efficacy of the 5′-UMP in younger, not older, subjects. Muscle atrophy and weakness typically manifest at a rate of approximately 10% every decade between the ages of 20 and 80 (44). This age-related decline in muscle mass and strength, known as “sarcopenia,” poses a significant societal challenge in aging populations, contributing to increased health and medical burdens (45, 46). Sarcopenia develops gradually over the long term with aging, characterized by a higher rate of atrophy in fast-twitch fibers than in slow-twitch fibers and a reduction in the number of muscle fibers (47). This distinguishes sarcopenia from short-term disuse muscle atrophy. Notably, muscle atrophy in the elderly results not only from aging but also from factors such as disuse (e.g., bed rest, inactivity), illness, and malnutrition (48, 49). Elderly individuals with low physical activity can experience up to 28% muscle atrophy in 1 year (50), underscoring the significant impact of reduced physical activity on muscle atrophy. It is necessary to investigate the efficacy of UMP in the elderly population in the future to reduce muscle atrophy in the elderly, especially as physical activity decreases.

This study has certain limitations. Participants were instructed to maintain their usual eating habits, but their diets were not monitored. Muscle atrophy was experimentally induced by detraining following resistance training-induced muscle hypertrophy, but the precise mechanism of detraining-induced muscle atrophy remains unclear (51). This study did not assess the expression or protein turnover of atrogin-1 or PGC-1α, necessitating further investigation into how 5′-UMP inhibits muscle atrophy during detraining. Additionally, the study did not examine the effect of 5′-UMP on muscle strength, possibly due to short 2-week detraining period. Long-term observations are warranted to clarify the effects of 5′-UMP on detraining-induced muscle strength loss. Further research should also explore the effects of 5′-UMP on disuse muscle atrophy resulting from joint immobilization or bed rest.

In conclusion, 5′-UMP may prevent early muscle atrophy due to the detraining after the hypertrophy in healthy men, although the mechanism to prevent muscle atrophy was unclear. UMP is also expected to prevent early muscle atrophy in athletes after injury and possibly in the elderly. However, because the effect was observed in healthy young men, this finding is not currently applicable to the elderly. Therefore, further research with biomolecular methods on biopsy samples, and different subjects and conditions is warranted.

## Data Availability

The raw data supporting the conclusions of this article will be made available by the authors, without undue reservation.
