# Supplementary material for: 5′-UMP inhibited muscle atrophy due to detraining: a randomized, double-blinded, placebo-controlled, parallel-group comparative study
Source: Front Sports Act Living. 2024 Jul 15;6:1403215. doi: 10.3389/fspor.2024.1403215 (PMC11284071; doi:10.3389/fspor.2024.1403215)
Supplement: Supplementary file 1 [file Datasheet1.docx]

**
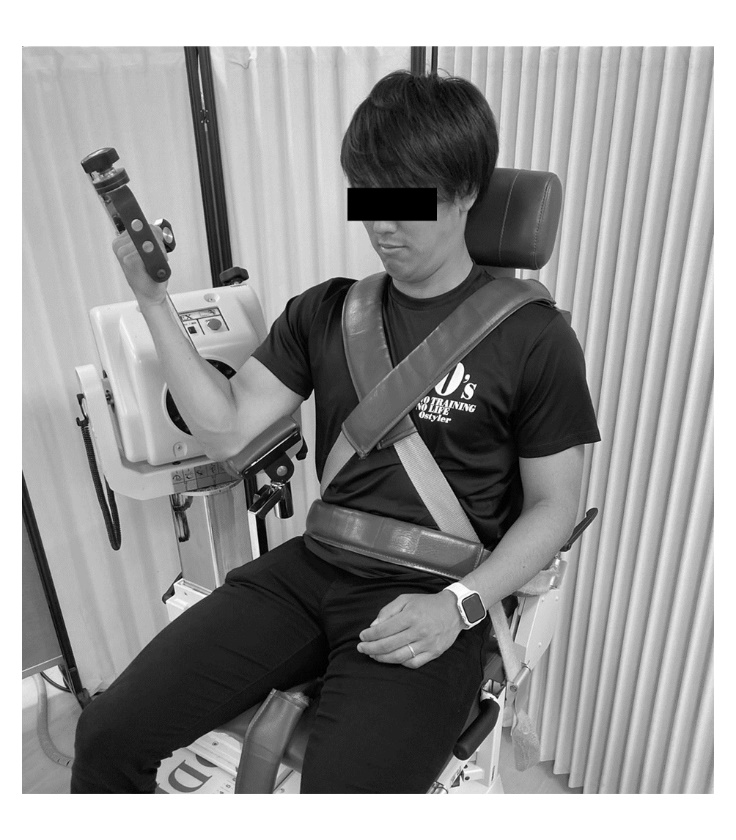
**

**Supplemental figure 1.** Biodex setting.


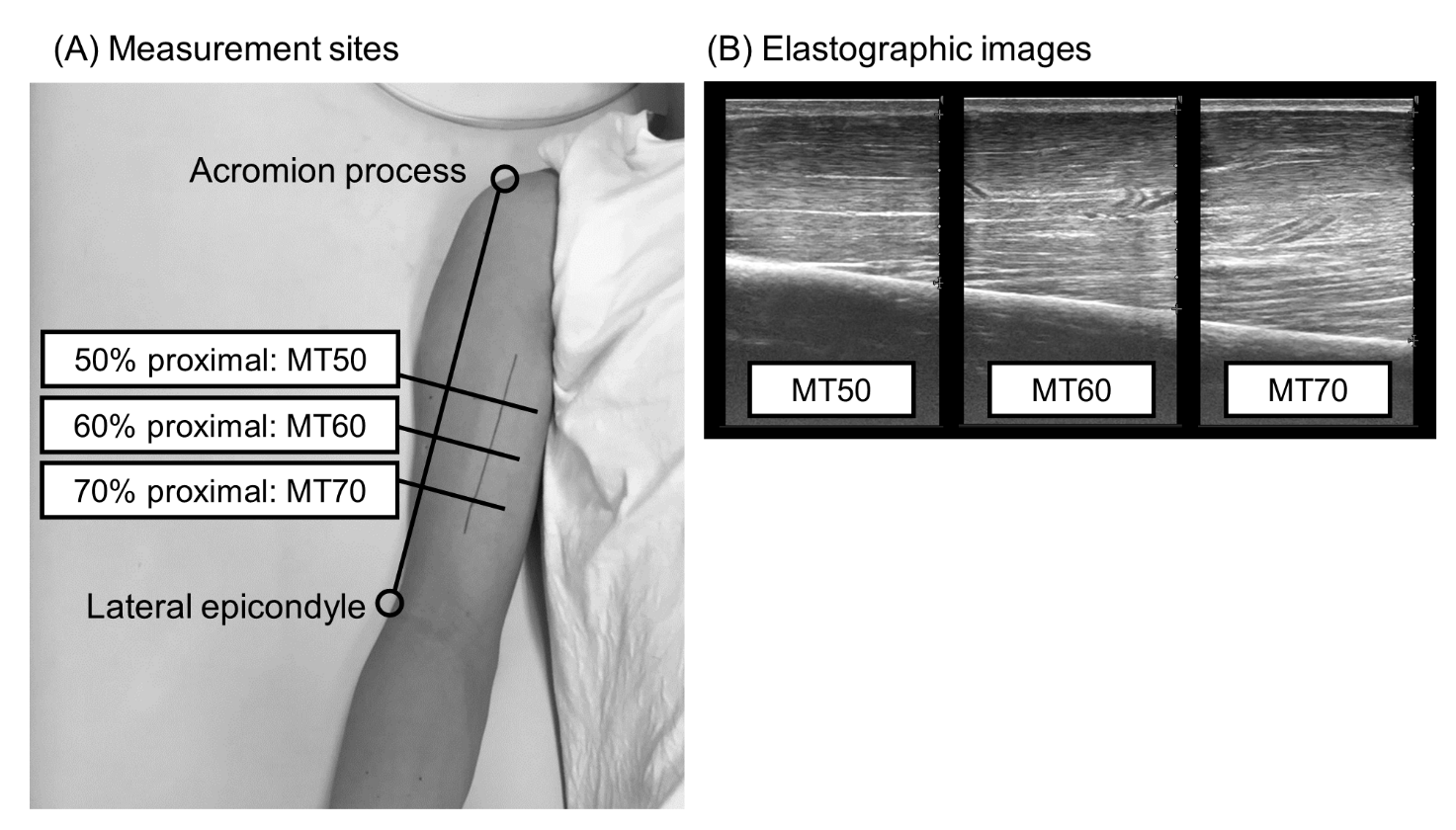


**Supplemental figure 2.** Measurement sites (A) and elastographic images (B) of muscle thickness. MT50, MT60, and MT70 are the sites at 50%, 60%, and 70%, respectively, of the distal part of the brachial length (from the acromion to the lateral epicondyle).

**Supplemental Table.** Changes in muscle strength and thickness due to resistance training

|  | ***n*** | **Before-training** | **After-training** | ***p*** |
| --- | --- | --- | --- | --- |
| Muscle strength (N·m) | 21 | 55.2 ± 11.6 | 63.6 ± 13.0 | 0.002 * |
| Muscle thickness (mm) |  |  |  |  |
| MT50 | 21 | 27.4 ± 3.0 | 29.1 ± 2.6 | < 0.001 * |
| MT60 | 21 | 31.9 ± 3.1 | 33.8 ± 2.9 | < 0.001 * |
| MT70 | 21 | 33.6 ± 3.1 | 35.8 ± 2.9 | < 0.001 * |
| Data are expressed as mean ± SD. * *p* < 0.05 | | | | |
